# Patient Pathway Aggregation – building on a firm foundation

**DOI:** 10.1186/1472-6963-11-S1-A7

**Published:** 2011-10-19

**Authors:** P Monteith

**Affiliations:** 1 NHS Information Centre, Trevelyan Square, Leeds, LS1 6AE, UK

## Introduction

Healthcare Resource Groups (HRGs) are the mechanism by which patient activity is classified according to Casemix in England. They are derived from care-activity data, primarily ICD-10 diagnosis codes and the United Kingdom’s OPCS-4 intervention and procedure codes, recorded in local hospital systems. Care events are recorded in standard datasets and processed through the HRG4 grouping algorithm to assign appropriate HRGs for each event.

HRGs are the primary funding mechanism for acute care in the English National Health Service (NHS) under the Department of Health’s Payment by Results (PbR) national policy. In the 2011/12 financial year, this covers admitted patient care, outpatient procedures and emergency medicine, with a total estimated expenditure of £30 billion.

As a precursor to calculating the national tariff for HRG4 “currencies”, where a “currency” is defined in the Department of Health’s PbR Guidance for 2011-12 as “the unit of healthcare for which a payment is made”, the Department collects annual cost data (Reference Costs) from every NHS provider of care. It uses this data as the basis for setting a national tariff and its related price.

The change in Government in the UK in May 2010 has resulted in a transformation in intended healthcare policy, with planned changes including the responsibility for commissioning of NHS and Specialist Services being transferred to Clinical Consortia and the newly established NHS Commissioning Board. At the same time, the State’s role in direct financial management is expected to be reduced as responsibility for national price setting under the current Department of Health Payment by Results (PbR) policy transfers to Monitor, currently an independent regulator.

As highlighted by the proposed Health Bill for 2011, the desire to move to outcome-based payments for healthcare based on patient pathways that are informed by clinical and financial best practice has not waned. In addition, the renewed emphasis on the patient journey, rather than its constituent parts, has led the Casemix team to reconsider the HRG4 classification in light of the new commissioner audience.

## Methods

In keeping with the fundamental principles of a Casemix classification being manageable in number, while retaining and indeed pursuing clinical relevance, the NHS Information Centre’s Casemix team recognises the inherent tension between the level of specificity required in a classification to effectively deliver and monitor healthcare provision and performance, and that required to commission healthcare services for a targeted population at the patient level. If a healthcare provider necessarily needs to understand service inputs in order to maximise efficiency and quality, yet ultimately minimise costs, a commissioner will and arguably should adopt a healthcare output, if not a healthcare outcome, perspective.

Previous attempts at developing patient pathways as a mechanism for funding healthcare in England have, however, been compromised by an inability to identify the cost of the component elements of healthcare contained therein, or at least on a consistent basis and applicable nationally. They have also been hampered by a lack of available standardised data beyond the traditional hospital setting, especially where care is transferred beyond the hospital and into the community, or to another healthcare provider.

As a result of the divergent classification requirements of healthcare provider and healthcare commissioner in the new NHS, coupled with a need to provide a pathway funding solution for costing and ultimately pricing identified pathways of care, the members of the Casemix team are investigating Patient Pathway Groups (PPGs) that can be assembled from the HRG4 classification.

While this approach has many advantages, those most notable include the ability to:

• Use national Reference Costs at an HRG4 level, including those for unbundled events additional to the core care episode, to provide a variable ‘value’ unit for the patient pathway.

• Base the elements of proposed pathway groups upon robust, clinically endorsed HRGs.

• Adopt an incremental and modular approach to development so that existing datasets can be utilised to provide relatively “quick wins”, with the option to extend the pathway to new settings and service areas as more data become available over time.

## Results

Early findings indicate that in all likelihood PPGs will utilise selective diagnosis entry criteria with event-based pathway modifiers to provide three levels of patient pathway stratification covering routine to complex care, although this has yet to be fully evaluated. Pilot results for a number of pathways are expected in autumn 2011.

## Conclusions

What is clear is that PPGs offer the possibility of providing a sophisticated aggregate commissioning currency for healthcare that overlays and builds upon the comprehensive HRG4 classification that remains pivotal to provider-level costing.

